# Sturge–Weber Syndrome: A Narrative Review of Clinical Presentation and Updates on Management

**DOI:** 10.3390/jcm14072182

**Published:** 2025-03-22

**Authors:** Aarnav D. Shah, Peter Alexieff, Priyamvada Tatachar

**Affiliations:** 1 Lake Forest Academy, Lake Forest, IL 60045, USA; 2 Ann & Robert H Lurie Children’s Hospital of Chicago, Chicago, IL 60611, USA; palexieff@luriechildrens.org (P.A.); ptatachar@luriechildrens.org (P.T.); 3 Northwestern University Feinberg School of Medicine, Chicago, IL 60611, USA

**Keywords:** Sturge–Weber syndrome, port wine birthmark, drug-resistant epilepsy, hemispherectomy, glaucoma

## Abstract

Sturge–Weber Syndrome (SWS) is a rare neurocutaneous disorder caused by a somatic nonsynonymous mosaic mutation most commonly in the GNAQ gene (G protein guanine Nucleotide-binding protein Alpha subunit q). SWS is characterized by capillary-venous malformations in the brain and eyes and a characteristic facial port wine (PW) birthmark (previously called port wine stain/PWS) in the head/neck region. Clinical manifestations vary and include epilepsy, stroke-like episodes, migraine headaches, cognitive delays, glaucoma, ocular vascular anomalies, heterochromia of the iris, visual field defects, and endocrine disorders like growth hormone deficiency or central hypothyroidism. The pathognomonic findings seen in neuroimaging with magnetic resonance imaging (MRI) include the presence of unilateral intracranial leptomeningeal angiomatosis, typically ipsilateral to the facial birthmark. SWS does not currently have a definitive cure, and management strategies focus on symptomatic management such as anti-seizure medications, limited surgical resection of the epileptogenic tissue or hemispherectomy for cases of drug-resistant epilepsy (DRE), selective photo-thermolysis of the PWS using a pulsed dye laser, and the medical and/or surgical management of glaucoma. In addition to these symptomatic treatments, the use of preventive, modifying, or stabilizing treatments like low-dose aspirin in reducing the frequency and severity of seizures and stroke-like events and the use of newer therapies like cannabidiols and mTOR inhibitors are being reviewed and have shown promising early results. This comprehensive narrative review summarizes the current literature on clinical management strategies, ongoing research studies, and future directions in the diagnosis and management of SWS.

## 1. Introduction

Sturge–Weber Syndrome (SWS), or encephalotrigeminal angiomatosis, is a rare neurocutaneous disorder. It occurs sporadically worldwide, seen equally in males and females and in all races and ethnicities globally [1]. SWS is most commonly attributed to postzygotic somatic mutations in the G protein guanine Nucleotide-binding protein Alpha subunit q (GNAQ gene), with recent research also implicating sporadic mutations in the *GNA11* and GNB2 genes. The hallmark features of this condition are as follows: facial port wine birthmark (PW, which used to be referred to as “Port Wine Stain” but is now referred to as the more positively connotated “Port Wine Birthmark) on the forehead and upper eyelid, typically seen on one side of the face; leptomeningeal angiomatosis, characterized by abnormal blood vessels on the surface of the brain; and underlying cortical atrophy, with calcifications typically also ipsilateral to the facial port wine birthmark. SWS severely impacts the quality of life of patients. Understanding current and potential future treatment methods is essential to provide the best care for those affected.

## 2. Epidemiology and Outcomes

SWS is the third most common neurocutaneous syndrome following neurofibromatosis and tuberous sclerosis complex, with an estimated prevalence of 1 in 20,000–50,000 live births with no known gender or racial predilection [2]. An observational study evaluating 18 years of data conducted by the Korean National Health Insurance revealed the incidence rate of SWS to be approximately 3.08 per 100,000 people per year [2,3]. In the United States, a study from Minnesota showed an incidence of 0.19/100,000/year [2]. A study evaluating the incidence of rare epilepsies showed an incidence of 1 in 40,900 for epilepsy related to SWS, which is higher than prior estimates [4].

Although there are no published data, it is believed that SWS does not affect a patient’s life expectancy; however, the symptoms can severely impact quality of life. The severity of neurological signs such as epilepsy, hemiparesis, and mental delay are prognostic factors for the severity of SWS in the patient. However, prognosis is extremely variable [5]. The disease course is highly variable with respect to the presence, onset, and intractability of epilepsy; motor dysfunction, and neurocognitive impairment. The early onset of catastrophic seizures is a bad prognostic factor for cognitive and motor deficit [6]. Comparing outcomes and mortality to another neurocutaneous disorder, neurofibromatosis (NF), a study found that the mortality due to presumed NF compared to the total U.S. mortality had a prevalence of 1/8700, which is approximately one-third to one-half of NF prevalence [7]. NF typically results in vascular conditions and increases the risk of gastrointestinal stromal tumors, which cause a greater mortality rate.

## 3. Genetics and Etiopathogenesis

The most common mutation associated with SWS is the R183Q mutation in the GNAQ gene located on chromosome 9 (9q21). *GNAQ* encodes Gαq, an alpha subunit of heterotrimeric G proteins. G proteins are a family of membrane-bound guanosine triphosphatases (GTPase) that transmit signaling from transmembrane G-protein coupled receptors. In particular, the R183Q mutation is believed to impair the hydrogen bond formation between the *R183* residue and GDP molecule, destabilizing the inactive GDP-bond conformation. There is believed to be a downstream activation of the Ras/Raf/MEK/ERK pathway as well as hyperactivation of the mTOR activity [8,9]. Recently, somatic gain of function mutations in GNA11 and GNB2 genes (p.Lys78Glu) have been associated with a distinct phenotypic variants of SWS. These mutations are thought to affect downstream MAPK signaling and YAP (yes-associated protein) in the Hippo signaling pathway, respectively [9]. These mutations are believed to cause abnormal proliferative capillary overgrowth and abnormal endothelial cells in blood vessels [2].

These gain-of-function mutations are translated into activated endothelial cells with proliferative capillary overgrowth or differentiation impaired endothelial cells with progressive dilatation of immature venule-like vasculature.

SWS causes capillary vascular malformations in the leptomeninges and impaired superficial venous drainage pathways in the brain [10]. It is believed that these vessels may have increased permeability, which allows for protein and calcium to enter the parenchyma and crystallize. Over time, this calcification becomes prominent in addition to enlarged deep-draining vessels that compensate for the increased venous pressure caused by the capillary venous malformations [10]. Calcifications are seen typically after age 2 in the parietal or occipital regions and appear as gyriform on skull radiographs or computed tomography (CT) scans, earning the eponymous term “Tram track” calcifications. The brain’s involvement in SWS occurs ipsilateral to the location of the PW; however, in approximately 15% of patients, bilateral brain involvement is seen [1]. Patients with bilateral involvement have more severe disease presentation and progression with a more unfavorable prognosis [6,11].

Previously, all patients with a facial port wine stain were believed to have leptomeningeal involvement and SWS. However, it has been demonstrated that the area of highest risk is the forehead, which is demarcated laterally and inferiorly by the outer margins of the canthus of the eye (including the upper eyelid) to the top of the ears. Aside from the location, the risk of SWS is also higher with more extensive facial capillary malformations [12]. The bilateral PW birthmark is the dominant risk factor for bilateral brain involvement in SWS. One study demonstrated that 42.4% of patients with bilateral PWS had bilateral brain involvement compared to 4.8% with bilateral brain involvement with a unilateral PW birthmark.

There have not been maternal risk factors identified in the development of SWS [13].

GNA11 mutations have been reported to have a more ambiguous involvement associated with a violaceous color of their PW birthmark, limb hypertrophy, and an overall milder neurological course [14].

## 4. Clinical Features

### 4.1. Neurological Abnormalities

Neurological manifestations are the most common symptoms in patients with SWS and include seizures and epilepsy, stroke-like episodes, migraines, and cognitive and psychomotor delays.

#### 4.1.1. Seizures and Drug-Resistant Epilepsy

Epilepsy is the most common and debilitating neurological feature in patients with SWS. Most patients present with seizures within the first year of life, with the median age of onset around 6 months. The predominant seizure types described are focal onset seizures, especially focal motor clonic seizures. Focal seizures often occur in clusters and may progress to focal status epilepticus [15]. Often, a postictal Todd’s paresis of variable duration is reported by caregivers following prolonged seizures or seizure clusters.

**Figure 1:**
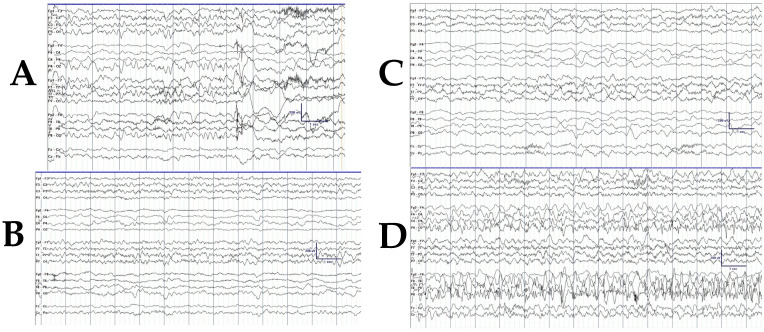
(**A**) Bipolar longitudinal montage of a 16-month-old patient with SWS demonstrating marked asymmetry with slowing attenuation on R. (**B**) Bipolar longitudinal montage of a 16-month-old with SWS demonstrating marked asymmetry with slowing attenuation on R. (**C**) Bipolar longitudinal montage of a 16-month-old with SWS in sleep, demonstrating preserved sleep spindles on the left. (**D**) Average referential montage of a 16-month-old showing seizure onset at P4 (right posterior region).

Electroencephalographic (EEG) findings in patients with focal onset seizures often show background asymmetry and focal slowing and/or voltage attenuation on the affected hemisphere, preserved organization and sleep architecture on the unaffected side, and seizure onset from the affected regions of the brain (Figure 1A–C). The focal seizures arise from the affected hemisphere (Figure 1) and can be electrographic-only or electroclinical.

Other seizure types seen in SWS are infantile spasms, myoclonic seizures, absence seizures, and atonic seizures [15]. The severity of epilepsy has been associated with the extent of the leptomeningeal enhancement, with bilateral and hemispheric cases showing earlier and more refractory epilepsy [16].

**Figure 2:**
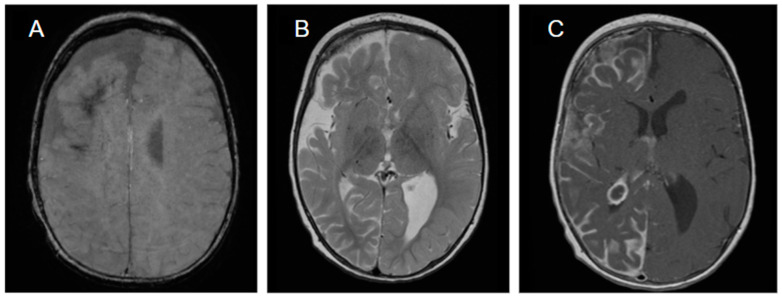
(**A**) Axial SWI imaging in a 16-month patient with right-sided SWS demonstrating hypo-intensity in the right frontal region, consistent with parenchymal calcification. (**B**) T2 axial imaging in a 16-month patient with right-sided SWS volume loss and hypo-intensity in white matter, most prominent in the right front region. (**C**) Post-contrast T1 axial imaging in a 16-month patient with right-sided SWS demonstrating diffuse right-sided enhancement over gyri, consistent with pial angiomatosis and a choroid plexus papilloma in the posterior horn of the right lateral ventricle (montage type: standard 10–20 montage; longitudinal bipolar or common average and reading sensitivity).

An example of brain magnetic resonance imaging (MRI) findings is shown in (Figure 2).

The cortex adjacent to the leptomeningeal angiomatosis is most often implicated as the source of seizures. The exact pathogenesis of epilepsy in these patients has not been elucidated. It is currently thought that cortical irritability results from impaired cerebral venous blood flow related to leptomeningeal vascular malformations. Long-standing cerebral ischemia may lead to focal neuronal loss, atrophy, and calcifications, contributing to ongoing epileptogenesis.

Cortical malformations like polymicrogyria and focal cortical dysplasia (FCD) type IIIc are commonly associated with SWS. Cortical neuronal dyslamination with neuronal cell loss and astrogliosis in areas adjacent to the leptomeningeal angiomatosis and loss of cortical lamination by NeuN immunohistochemistry has been described using histopathology [17].

#### 4.1.2. Stroke-like Episodes

A common presentation among SWS patients is stroke-like episodes with transient neurological deficits like hemiparesis or visual auras with migraines leading to unilateral weakness, typically lasting longer than 24 h. These symptoms occur between 6 months and 5 years of age, and head trauma is believed to be a strong trigger. Significant variability exists among these episodes with respect to the degree of weakness, time to recovery, and association with seizures. The pathogenesis is unclear, but is believed to be related to seizure activity, hypoxia, and capillary leakage. Aspirin at a dose of 3–5 mg/kg/day was associated with the lowered frequency and severity of stroke-like episodes [18].

#### 4.1.3. Migraines and Headaches

Headaches occur more frequently in patients with SWS than in the general population. Headaches are usually migrainous with or without visual auras, and may be associated with hemiparesis and stroke-like episodes. Migraines associated with SWS showed no gender differences and had an earlier age of onset. The median age at onset was 8 years, and the frequency averaged three headaches per month [15]. Clinical manifestations of these headaches in young children may also include episodes of vertigo, asthenia, and sleepiness. Late-onset hemiplegic migraines have also been reported [19]. Headaches were also found to be associated with glaucoma. The pathophysiology of headaches is possibly related to vascular flow and oligemia, but this is not fully elucidated. Vomiting, fever, and dehydration can create a hyperviscous intravascular state, increasing the risk of headache and thrombosis.

Headaches secondary to life-threatening causes in SWS are very rare and are especially under-reported in the absence of other neurological signs and symptoms. In addition to improving sleep hygiene and hydration, and using acetaminophen or NSAIDs and antiemetics for acute attacks, access to prophylactic daily therapies like lamotrigine, topiramate, depakote, or gabapentin is recommended. Triptans have been found to be safe to use in aborting acute migraine attacks in patients with SWS [20].

#### 4.1.4. Intellectual Disability and Behavioral Problems

**Figure 3:**
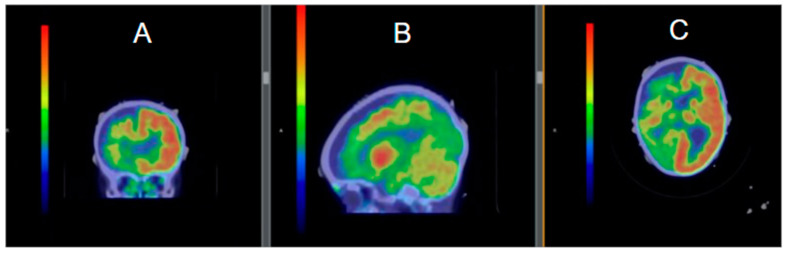
PET scan in a 16m patient with right-sided Sturge–Weber Syndrome in (**A**) coronal, (**B**) sagittal, and (**C**) axial views, demonstrating significant hypometabolism throughout the R hemisphere.

Intellectual disability developed in 60% of SWS patients and was severe in about 33% of patients [21]. Another study found the rate of developmental delay to be 43% in SWS patients [22]. Typically, SWS patients develop normally for several months before developmental delay manifests, but the timeline of development is highly variable. Behavioral problems are more common in SWS patients than in siblings without SWS [23]. A study conducted found that within a cohort of SWS patients, 24% were diagnosed with autism spectrum disorder and 45% showed evidence of social communication difficulties [24]. In total, 50% have significant behavioral difficulties and 26% have difficulties with sleep [24]. In bilateral SWS, the degree of hemiparesis and developmental delay may be associated with the extent of bilateral hypometabolism measured using positron emission tomography (PET) (Figure 3). Seizure control was also associated with better developmental outcomes in this population. Furthermore, this study suggests that PET studies showed a larger area of hypometabolism than the lesions identified on computed tomography, indicating a larger zone of functional deficit [6].

### 4.2. Cutaneous Manifestations

Port wine (PW) birthmarks (also known as nevus flammeus) are common congenital capillary malformations of the skin which appear as flat pink to red macules typically seen in the head and neck area. They are common in the general population and are found in about 3 out of 1000 births. However, only 3%–6% of these patients have associated eye findings, brain involvement, and neurological deficits leading to the diagnosis of SWS [11,25,26]. Hennedige et al. [25] reported that, among 874 patients with facial port wine stains, features of SWS were seen in 30 patients. Another study found that 13/30 patients born with a PW birthmark had SWS with leptomeningeal involvement [27]. These studies indicate that early screening for SWS should be conducted on patients born with a PW birthmark. The location of these birthmarks can help predict the risk of brain and eye involvement. These vascular malformations can be unilateral, bilateral, or midline in location. New research suggests that the distribution is more related to the embryological vascular patterns than the trigeminal nerve distribution, as previously believed. The biggest independent risk for associated ocular and brain findings and SWS are seen with PW birthmarks in the forehead distribution, and severity is correlated with bilateral distribution or extensive unilateral distribution involving the mandibular area.

### 4.3. Ocular Manifestations

Ocular manifestations occur in over half of patients with SWS and are an important clue to initial diagnosis when a facial PW birthmark is present. The associated risk of leptomeningeal or ocular involvement depends on the extent and location of the capillary malformations (CM), as discussed above, and ranges from 7% to 28%. The two most common manifestations include glaucoma and the presence of choroidal hemangiomas. Glaucoma, most commonly in an open-angle form, is seen in 30–70% of SWS patients, with a large proportion occurring in early infancy and childhood between 0 and 3 years of life (60% early onset and 40% late onset presentation). Symptoms include vision loss, development of dilated conjunctival vessels, eye pain, excessive tearing, and, in infants, eye enlargement [9]. If untreated, glaucoma can cause vision loss [9]. Therefore, all infants born with a facial or eyelid PW birthmark should undergo an ophthalmological exam measuring intraocular pressure (IOP) with frequent reassessments. The other ocular manifestations include dilated episcleral vessels, retinal detachment, diffuse choroidal hemangioma, strabismus, and refractive errors [2].

### 4.4. Endocrine Abnormalities

In addition to the more common presentations mentioned above, growth and constitutional abnormalities can be seen in patients with SWS related to endocrine dysfunction. Growth hormone deficiency was found to be 18 times more likely to occur in SWS patients compared to the general population (0.54% vs. 0.03%), and may be the etiology of impaired growth noted in children with SWS [28]. Another study found 2.4% of SWS patients to have central hypothyroidism, much higher than the general population [29].

## 5. Diagnosis and Management

### 5.1. Diagnosis

There is no specific treatment for SWS, and management is based on treating manifested symptoms. Given the multisystem involvement, early diagnosis is key for effective management of the clinical and psychosocial aspects of SWS. Recent consensus statements have been published with recommendations and clinical practice guidelines for the diagnosis, surveillance, and multidisciplinary management of SWS [30,31,32,33,34].

Key diagnostic criteria involve two out of the following three features: a characteristic facial port wine birthmark (PWB) in the high-risk distribution, vascular malformations of the eye, and brain MRI findings of leptomeningeal angiomatosis. If the newborn is deemed at risk for SWS, it should be referred for evaluation by an ophthalmologist and neurologist experienced in the management of SWS. An eye exam and evaluation of intraocular pressure by an ophthalmologist is critical when evaluating glaucoma at diagnosis. The timing of neuroimaging at diagnosis depends on the extent of the port wine birthmark and neurological symptoms. Brain MRI with and without gadolinium contrast with susceptibility (SWI) and diffusion (DWI) weighted sequences is recommended. It is important to note that imaging may be false-negative initially due to incomplete myelination, and a full imaging study is recommended after 1 year of age. A screening MRI should be considered in extensive PW birthmarks if presymptomatic treatment is considered. The use of repeated computed tomography (CT scans) should be avoided. Routine follow-up neuroimaging is not recommended in children with established SWS unless there is concern for stroke where rapid sequences with DWI are preferred.

### 5.2. Epilepsy Management

Treatment of seizures can include. presymptomatic management, or symptomatic management with anti-seizure medications or surgical management of intractable epilepsy.

#### 5.2.1. Medical Management of Seizures and Epilepsy

As of the current date, there is no consensus or universally accepted guideline on how soon anti-seizure medications (ASMs) should be started in presymptomatic patients for prophylactic therapy. Neither is there any preference between the commonly used anticonvulsants available. Based on several studies, the most commonly used anti-seizure medications used to treat epilepsy in patients with SWS include levetiracetam (48.1% in one study), oxcarbazepine (39.9%), and phenobarbital (14.9%); Lamotrigine (8.2%), lacosamide (7.8%), and clobazam (2.6%) were the second-line ASMs used [35].

Only 37.7% of patients were on monotherapy (only one medication), while the rest were on polytherapy with ≥2 anti-seizure medications. Of the patients on monotherapy, oxcarbazepine was the most frequently used, followed by levetiracetam and carbamazepine [35]. Levetiracetam was the most common ASM used in a multi-drug regimen due to ease of titration and few drug interactions.

The response to anti-seizure therapy is mixed in patients with SWS. A study of anticonvulsant efficacy for SWS patients found that 56.5% of patients with oxcarbazepine or carbamazepine history were seizure-free, compared to 31.4% who had not been on these ASMs. The same study found that 61.3% of patients taking the anticonvulsant medication were seizure-free, compared to 28.6% who were not taking any medication [36].

In patients with concomitant migraines, treatment with lamotrigine, valproic acid, and topiramate has been used. Topiramate has not been shown to cause or worsen glaucoma when used in patients with SWS.

Standard anticonvulsants are typically used first to treat epilepsy in SWS, with varying success. In patients with epilepsy, refractory to medications, alternative therapies like vagus nerve stimulators and a ketogenic diet can sometimes be successful [37].

#### 5.2.2. Surgical Management of Drug-Resistant Epilepsy

Surgical management, such as a focal resection or hemispherectomy/hemispherectomy, is a option for cases with drug-resistant and poorly controlled epilepsy and in patients with large or hemispheric involvement. Drug-resistant epilepsy is defined as poor seizure control despite optimal doses of two ASMs [38]. One study found that, of the 90 patients who underwent surgery, 83.33% achieved seizure freedom, 44.44% achieved favorable cognitive outcomes, and 43.33% achieved favorable motor outcomes. Additionally, modified hemispherectomy or hemispherectomy (either functional or minimally invasive) was safer than anatomical hemispherectomy [39]. Epilepsy surgery is less often considered in bilateral SWS, as there may be bilateral epileptogenic seizure foci. However, in cases with a clear predominant epileptogenic focus, several case studies have demonstrated successful surgical outcomes with a near-total elimination of seizures. These patients made developmental gains, but remained delayed [40]. Both of these case reports reported a functional left hemispherectomy. Focal resection or hemispherectomy are also options, especially in refractory epilepsy with hemiparesis [38]. Improvements in cognitive and motor outcomes have been reported postoperatively in addition to significant improvements in seizure burden. In a recent review [41], Mozaffari et al. performed a meta-analysis of 18 studies with 108 patients. The median age at seizure onset was 4.5 months, and 85 patients (78.7%) were seizure-free at the last follow-up (median: 72 months), with no statistically significant differences between hemispherectomies and tailored focal resections. In a large multicenter retrospective observational study spanning 20 years, Wang et al. [42] found that early surgical treatment (under the age of 2 years) led to better cognitive and motor outcomes after hemispheric surgeries.

#### 5.2.3. Additional Management Strategies in Seizure and Epilepsy

In addition to the abovementioned anti-seizure medications and surgical treatment, low-dose aspirin has been used to prevent stroke-like episodes and has even been found to reduce seizures in some cohorts, although larger controlled studies are needed [18]. It is thought that aspirin can combat the chronic hypoperfusion and subsequent neuronal injury present in SWS [43].

Presymptomatic treatment: Although there are no randomized studies to support presymptomatic anti-seizure therapy for SWS, this approach has been used to prevent epileptic kindling and slow the cumulative damage to the brain that multiple severe seizures have been shown to cause. Small prospective studies have shown the benefits of this approach, but larger controlled studies are still needed [43].

### 5.3. Glaucoma and Ocular Issues

Glaucoma is treated by lowering intraocular pressure, which can be performed using ocular drops, which lower eye pressure, and glaucoma surgery if eye drops fail to alleviate the condition [11,44]. Karaconji et al. found varying success using trabeculotomy and Baer veldt tube shunt procedures in a study conducted from 2003 to 2016 [45]. A study comparing the effectiveness of first-line medications in treating open-angle glaucoma found that the most to least effective drugs (as measured by decrease in pressure in millimeters of mercury after 3 months) is as follows: bimatoprost 5.61 (4.94; 6.29), latanoprost 4.85 (4.24; 5.46), travoprost 4.83 (4.12; 5.54), levobunolol 4.51 (3.85; 5.24), tafluprost 4.37 (2.94; 5.83), timolol 3.70 (3.16; 4.24), brimonidine 3.59 (2.89; 4.29), carteolol 3.44 (2.42; 4.46), levobetaxolol 2.56 (1.52; 3.62), apraclonidine 2.52 (0.94; 4.11), dorzolamide 2.49 (1.85; 3.13), brinzolamide 2.42 (1.62; 3.23), betaxolol 2.24 (1.59; 2.88), and unoprostone 1.91 (1.15; 2.67) [46]. Lifelong monitoring for the development of glaucoma and other ocular complications related to SWS should be exercised even if complications do not appear early on [47].

### 5.4. Port Wine Birthmark

**Figure 4:**
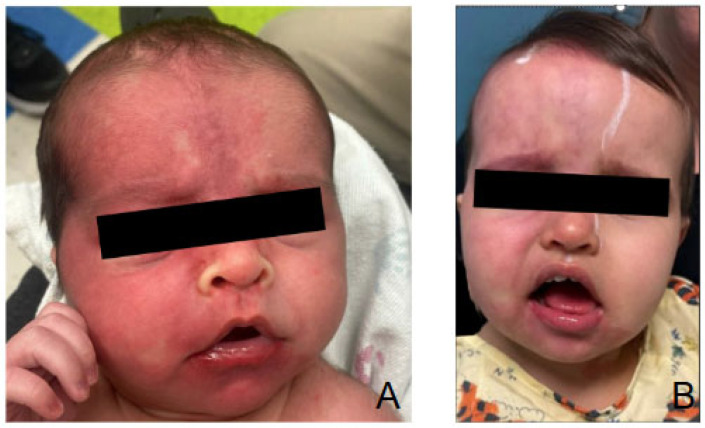
Port wine stain involving the right side of the face seen in a newborn (**A**). Same patient at 16 months of age (**B**) following six pulse dye laser treatments.

The PW birthmark is typically removed with pulse dye laser (PDL) treatment [33] (Figure 4). This treatment is used regardless of lesion size, location, or color, and the treatment can be performed safely on patients of all ages when performed by experienced physicians [33]. The treatment is repeated several times, as the port wine birthmark can reappear and darken over time. However, PDL is not always successful: a study found that, of 30 patients with diagnosed SWS, only 45% of syndromal patients and 55% of nonsyndromic patients had a satisfactory outcome in color and size reduction in the birthmark [25].

Topical treatments also exist; however, their success rate is variable. A study found that the treatment showed significant improvement in one out of three studies using topical sirolimus, but the other two reported no improvement [48]. Two studies examined the effectiveness of timolol and reported no change in the PW birthmark appearance compared to placebo [48]. One study examined the effectiveness of imiquimod and found that adding 5% adjuvant imiquimod cream led to significant improvements [48]. In these studies, Imiquimod and Sirolimus led to mild cutaneous adverse events, while timolol caused no side effects [48]. Systemic sirolimus has also been studied as a treatment for PW birthmarks. While systemic sirolimus did not significantly improve the PW birthmark, there were improvements in processing speed [49]. Another study found that the use of PDL and topical rapamycin showed statistically significant improvements compared to using PDL alone, rapamycin alone, and no treatment [50].

### 5.5. Measures to Improve Quality of Life

The combination of clinical manifestations in SWS can significantly impact quality of life (QoL). The NIH Quality of Life in Neurological Disorders (Neuro-QoL) is a useful tool for measuring neurological conditions’ physical, mental, and social effects. It can also be used to assess QoL in SWS patients. Prior studies have shown a negative correlation between the extent of PW birthmark size, brain and eye involvement, and neuro-QoL and cognitive function [51]. Therefore, as signs/symptoms of SWS become more diffuse, lower QoL is seen in those with bilateral involvement.

Additionally, there is also an increased risk of suicidal ideation in SWS in comparison to other neurological disorders [52]. Therefore, careful evaluation of these factors and their effects on QoL is important. Recently, an SWS-neurological rating score (SWS-NRS) was developed to assess the cumulative neurological impairment of SWS patients [53]. This scoring system is based on the observed visual defects, frequency of seizures, extent of hemiparesis episodes, and degree of cognitive dysfunction. This scoring system can be useful in monitoring clinical improvement when providing treatment or therapies for various neurological manifestations as an adjunct to standard monitoring tests such as MRI and electroencephalograms (EEG) [35,54,55].

## 6. Newer Therapies

### 6.1. Cannabidiol (CBD)

There is a growing body of literature on potential benefits in the management of epilepsy and seizure disorders shown in mouse models [56,57,58]. CBD can potentially attenuate seizure activity by inhibiting a G Protein-coupled Receptor (GPR55) [36,59]. In human studies, CBD has been approved for the treatment of seizures in Lennox–Gastaut syndrome and Dravet syndrome, and its use is now being expanded in other seizure disorders, including SWS [60,61,62]. Limited data from two small cohort studies suggest that CBD use was associated with reduced seizure activity, improved cognitive function, speech and communication, physical capability, and noticeable improvement in SWS Neuroscore and patient-reported QoL [31,63].

A recently concluded prospective study [31] with SWS patients with brain involvement, controlled seizures, and cognitive impairment gave CBD for 6 months and showed significant improvement in SWS neuro score, patient-reported QOL, anxiety, and emotional regulation, and reports of bimanual ability use.

### 6.2. mTOR Inhibitors

The mTOR pathway controls the expression of VEGF (Vascular Endothelial Growth Factor), and VEGF controls angiogenesis (growth of blood vessels). The mTOR pathway undergoes hyperactivity in Sturge–Weber patients, so it is theorized that using mTOR Inhibitors will stop this hyperactivity and abnormal blood vessel development. A recent open-label prospective study with ten subjects with SWS using oral sirolimus for 6 months showed improvement in QoL, cognitive function, and processing speed [49]. In addition to this cognitive improvement, Sirolimus therapy also controlled epileptic symptoms in drug-resistant epilepsy [64]. There were also improvements related to the cutaneous symptoms with reduced PW pigmentation, improved appearance of facial hemihypertrophy, and decreased soft tissue overgrowth [65,66]. Although these early results are promising, these studies have been conducted on very small SWS cohort sizes, and further studies are needed to evaluate the use of mTOR inhibitors in SWS patients.

## 7. Conclusions and Future Directions

Sturge–Weber Syndrome is the third most common neurocutaneous disorder. The neurological manifestations of seizure disorder, stroke-like symptoms, headaches, and cognitive impairment, along with other ocular and cutaneous involvement, have a significant impact on the quality of life of these patients. Epilepsy in SWS is often drug-resistant. Early consideration of surgical management is recommended in these medication-refractory patients. In addition, novel therapies such as CBD and mTOR inhibitors have shown promising early results, but need to be further evaluated in terms of their safety and efficacy. It is encouraging that these newer therapies have reduced seizure activity and improved cognitive function, speech, communication, and overall improvement in QoL. The effective management of patients with SWS depends on multidisciplinary coordinated care, including, but not limited to, primary care providers, neurologists, neurosurgeons, ophthalmologists, dermatologists, psychology and behavioral therapists, social workers, and rehabilitation medicine. Future studies should focus on evaluating first-line anti-seizure therapies and establishing standard-of-care guidelines, standardized neuroimaging protocols, improved brain mapping techniques for surgical procedures, the safety and efficacy of the CBD and mTOR inhibitors in the early and presymptomatic management of SWS, and strategies to improve overall QoL in SWS. As our understanding of the molecular mechanisms of SWS continues to improve, there is hope for more targeted and precise therapy in the treatment of this multisystem disorder.

## Abbreviations

ASM	anti-seizure medication
CBD	Cannabidiol
CM	capillary malformations
EEG	electroencephalography
GNAQ gene	G protein guanine Nucleotide-binding protein Alpha subunit q
GPR	G protein-coupled receptor
MRI	magnetic resonance imaging
mTOR	mechanistic Target of Rapamycin
NF	neurofibromatosis
PDL	pulsed dye laser
PW	port wine birthmark
QoL	quality of life
SWS	Sturge–Weber Syndrome
SWS-NRS	Sturge–Weber Syndrome Neurological Rating Score
VEGF	vascular endothelial growth factor
